# Sensation

**Published:** 1889-12

**Authors:** George W. Whitefield

**Affiliations:** Chicago


### SENSATION.[1]

BY GEORGE W. WHITEFIELD, M.D., D.D.S., CHICAGO.

This is a difficult and complex subject to treat, and one that from its very nature compels me to look to standard authorities before mapping out my course.[2] The subject may be dry, but still, to me, it has a subtle charm, pertaining, as it does, to the fundamental principle of learning. To understand the causation of sensation is to possess the key to the study of human nature,—almost the acme of human knowledge.

As an intelligent man reads and understands what is written, so a wise man reads the unwritten language of human thought expressed in pose, face, gesture, and action.

First of all, let us consider the lowest form of nerve-system that we possess knowledge of,—the centipede's. The creature is possessed of a great number of little brains, nerve-centres or ganglia, one to each of its many joints, to preside over each pair of legs. The action is like this: As each pair of legs touch is the ground, a sensory impulse is transmitted by the afferent nerves to the reflex centres, there to be so modified that it causes a motor impulse to be propagated along the motor-nerves to the muscles, causing them to act. That the reflex centres can act independently of a higher centre, or the true brain of the creature, can be illustrated by snipping off its head while in motion. It will continue to run until it meets an obstacle.

Animal life could not exist without the part played by reflex nerve-action. This is just as true of man as of the lower forms of life. In man the spinal cord may be considered as a downward prolongation of the brain, with which it is most intimately connected. It is composed of two parts,—an outer white portion, and an inner one of gray matter. The white portion is composed of bundles of

---

[1] Read before the Chicago Dental Club, September 23, 1889.

[2] Three years ago, when lecturing on pathology before dental students, I gave some lectures on sensation. From these lectures I have prepared the first part of this paper. At this late date I cannot give my authorities. I am especially indebted to the author of a pamphlet which I read at that time, but cannot recall the author's name. From it I gained much of the substructure of this paper.

nerve-fibres, for the transmission of nervous impulse in both directions. The gray portion is composed of highly-endowed nerve-cells, constituting ganglia or nerve-centres, which, under stimuli, have the power of transforming sensory into motor impulses. It will not be necessary for me to describe the anatomy of the spinal cord, but I will speak of the elements co-operating in the production of every physiological act,—the sensory nerves to convey towards the centre the impulse set in motion by external causes, the nerve-centre excited to active change by the stimulus received, and the motor or afferent nerves to transmit the energy transformed in the nerve-centres to the organ which in turn is excited to action.

The plan of nerve-action is simple if you comprehend these principles. Nerve-action can be compared to pulses, throbs, or explosions of nervous energy, according to the degree of action.

There is, arising under sensory stimulus, a pulse of excitement, and a discharge of energy, and then a subsidence followed by a never-ending quiescence, unless the centre is again stimulated from some source external to itself.

Please bear this in mind: there is first the pulse of excitement carried to the nerve-centre, followed by the explosion of nervous energy at the nerve-centre. As there can be no explosion of nervous energy without a prior sensory impulse from without, carried to the nerve-centre by the sensory or afferent nerves, the centres are not capable of self-stimulation. Thus you see what an important factor in animal life is sensation. In fact, the term sentient being is often employed as synonymous with animal life.

We have been speaking of sensory impulses. We will now consider what constitutes conscious sensation. Let me call your attention to the fact that anatomically the brain is a prolongation upward of the spinal cord. In other words, the brain is the enormously expanded portion of the cord, with its centre turned out. The cord in man is anatomically continuous, yet it is just as much the seat of reflex action as though it was, as in the centipede, composed of numerous anatomical separated nerve-cells or ganglia. Its continuity of structure facilitates the rapid transmission of sensation to and from itself, or to the higher nerve-centre,—the central organ of the whole nervous system. It is with the brain as a central nerve-organ, a congeries of differentiated and highly specialized nerve-centres,—a nerve-centre of nerve-centres,—that we have more especially to deal.

I have used the simple forms of reflex nerve-action to render more clear the phenomena of all nerve-action,—the pulse, throb, or explosions of energy, according to the amount of stimulation in the nerve-centres; the discharge of motor impulse to excite into action the organ to which it is sent.

An understanding of this will explain what takes place in the brain itself; for the brain is a nerve-centre,—with extraordinary endowments and of immense size, it is true, but it is a nerve-centre just the same. It acts only under stimuli, just like any other nerve-centre, even those of the centipede, but with this difference: all subordinate nerve-centres receive their stimulus from without, by the afferent or sensory nerves, unaided by the power of self-stimulation; not so the brain, for it is capable of self-stimulation,—that is, it receives stimuli from the direction of the external media through the five senses, and also from the direction of that unknown and unknowable something which we term the mind. But whether the stimulation comes by way of the senses or is self-generated matters not: the important fact remains that the effect produced is identical.

For instance, the thought of luscious fruit recalls a sensation of the odor and taste varying only in degree, not in kind, and the salivary glands are excited to activity. A sensory impulse, no matter what produces it, provided it originates outside of the sensorium, is first transmitted to its own nerve-centre or centres on its way to the sensorium, producing everywhere its appropriate reflexes, until finally it reaches the great nerve-centre, the brain, and we become conscious of the impulse by the throb or pulse of action, which has been produced in the unstable substance of the nerve-cells, just as in the minor reflex centres of the cord.

On the other hand, the changes are identical with the changes that take place when the stimulus is centrally initiated. Thus we see the brain is constantly subjected to these two diverse sources of stimulation,—viz., from the periphery by way of the senses, or from the mind.

A sensory impulse becomes a cognition in two ways,—by repetition and comparison, by the relation of differences. We are incapable of perceiving anything but by differences. It may be the difference of kind, degree, or time, but there must be a difference in one or more of these ways, or we fail to feel.

(To be continued.)

